# Food insecurity and health conditions in the Australian adult population: A nationally representative analysis

**DOI:** 10.1111/1747-0080.12907

**Published:** 2024-10-21

**Authors:** Jane M. Fry, Jeromey B. Temple, Ruth Williams

**Affiliations:** ^1^ School of Life and Medical Sciences and School of Health and Social Work University of Hertfordshire Hertfordshire UK; ^2^ Melbourne School of Population and Global Health University of Melbourne Melbourne Victoria Australia; ^3^ School of Population Health Curtin University Perth Western Australia Australia

**Keywords:** Australia, food insecurity, health, poverty

## Abstract

**Aim:**

This study aimed to identify key health condition correlates of food insecurity in Australia using nationally representative data.

**Methods:**

This cross‐sectional study used data from a large, nationally representative Australian survey that included questions on the dynamics of families and households, income, wealth, welfare, labour market activity (including unemployment and joblessness), life satisfaction and wellbeing. Binary logistic regression models of eight items of food insecurity measured the association between 17 health conditions and food insecurity while controlling for various demographic and socioeconomic variables. A zero‐inflated negative binomial model identified correlates of the number of food insecurity problems.

**Results:**

Prevalence of food insecurity ranged from 3% to 9% depending on the measure analysed. Individuals experiencing blackouts, fits or loss of consciousness were 2–6 times more likely to report food insecurity than other individuals. When including control variables and incorporating other health conditions, several conditions significantly increased probability of any food insecurity: sight problems; blackouts, fits or loss of consciousness; difficulty gripping things; nervous conditions; mental illness; and chronic or recurring pain.

**Conclusions:**

Detailed information on how health conditions are associated with different types of food insecurity was generated using population‐representative data, 17 sets of health conditions, and eight measures of food insecurity. Understanding connections between food insecurity and health conditions allows public health professionals to create effective, targeted and holistic interventions.

## 1 INTRODUCTION

Food insecurity is defined as ‘limited access to or availability of nutritious food or a limited/uncertain ability to acquire food in socially acceptable ways’.^1^ ^(p1840)^ Food insecurity is a problem for both low‐ and high‐income countries and, consistent with the Sustainable Development Goal of Zero Hunger, the United Nations Food and Agriculture Organization (FAO) encourages all countries to monitor food insecurity. In Australia, food insecurity has been estimated to affect about 3%–4% of the population based on a single‐item metric.^2^, ^3^, ^4^ The Food Insecurity Experience Scale was developed by the Food and Agriculture Organization and is an experience‐based measure for use across sociocultural contexts of household or individual food insecurity.^5^ In 2014, the Food and Agriculture Organization began collecting Food Insecurity Experience Scale data by leveraging the Gallup World Poll, which surveys nationally representative samples of the adult population annually in nearly 150 countries. This created the analytical protocols necessary to take the measurement global, making it possible to compare prevalence rates across countries and even sub‐national populations.^6^ The Food Insecurity Experience Scale has been used and validated in many communities such as in rural Bangladesh,^7^ in the League of Arab States,^8^ Sub‐Saharan Africa,^9^ The Bahamas,^10^ Malaysia.^11^ The 8‐item Food Insecurity Experience Scale allows for a more nuanced analysis of food insecurity, with items representing different levels of severity and ranging from ‘worry’ (mild) through to going hungry (severe).^12^ Analysis of these items reveals considerable heterogeneity in estimates of food insecurity prevalence, ranging from about 3% of adults who ran out of food to about 9% reporting limited variety in food intake in 2020.^13^ It should be noted that the Australian bushfires in 2019 and global pandemic in 2020 may have contributed to food insecurity.

Previous studies have found food insecurity to be associated with poor general health,^14^, ^15^, ^16^ poor mental health,^17^ limitations^18^ and disability.^17^ More specifically, food insecurity among adults has been linked to mental or cognitive disability (social/behavioural, memory, learning, understanding/concentration)^17^ and functional limitations or impaired activities of daily living, such as getting in/out of chairs, feeding, getting dressed, bathing and toileting.^16^, ^18^, ^19^ There is also evidence linking food insecurity with particular health conditions. For example, chronic or recurring pain,^18^, ^20^, ^21^ diabetes (either type),^16^, ^18^, ^22^ coronary heart disease,^16^, ^18^ heart attack,^18^ hypertension,^16^, ^18^ stroke,^18^ anxiety and depression,^16^, ^21^, ^23^ and uncontrolled asthma.^24^ Sight problems have also been associated with food insecurity. For example, in a study of US adults aged 50 years or over, food insecurity (whether marginal or very high) was associated with significantly higher odds of both self‐reported and presenting vision impairment, even when controlling for other sociodemographic and health factors.^25^ In a study of adults aged 50 years or over, food insecurity was negatively associated with self‐perceived and objectively measured hearing health.^26^

The aim of this study was to identify key health condition correlates of food insecurity in Australia using nationally representative data. This paper builds upon previous international and Australian studies by identifying the prevalence of food insecurity among Australian adults (aged 15 years or over) stratified by selection of health conditions. Associations between these health conditions and food insecurity are then explored to determine key health correlates of food insecurity. Finally, the paper presents results on key health conditions associated with the number of items on which individuals report food insecurity.

## 2 METHODS

Study data came from the Household, Income and Labour Dynamics in Australia (HILDA) Survey.^27^ HILDA is a large, nationally representative survey that has been tracking individuals annually since 2001. Survey topics include the dynamics of families and households, income, wealth, welfare, labour market activity (including unemployment and joblessness), life satisfaction and wellbeing. The scale of HILDA and its representativeness made it ideal for analysing detailed aspects of food insecurity at a population level. In 2020, HILDA surveyed over 17 000 individuals aged 15 years or over. Using HILDA's weights we estimated population‐wide results, taking into account non‐completion of the questionnaire. Watson and Wooden^28^ provide further details of HILDA.

Health conditions were identified using yes/no indicators for 17 health conditions included in HILDA: sight problems not corrected by glasses/lenses; hearing problems; speech problems; blackouts, fits or loss of consciousness; difficulty learning or understanding things; limited use of arms or fingers; difficulty gripping things; limited use of feet or legs; a nervous or emotional condition which requires treatment; any condition that restricts physical activity or physical work (e.g., back problems, migraines); any disfigurement or deformity; any mental illness which requires help or supervision; shortness of breath or difficulty breathing; chronic or recurring pain; long‐term effects as a result of a head injury, stroke or other brain damage; a long‐term condition or ailment which is still restrictive even though it is being treated or medication being taken for it; any other long‐term condition such as arthritis, asthma, heart disease, Alzheimer's disease, dementia, etc. Some health conditions were more common than others in the population, with prevalence ranging from 0.6% to 13%.

The 2020 data contained a new module on food insecurity, comprising eight questions: *During the last 12 months, was there a time when, because of a lack of money*:

- *You were worried you would not have enough food to eat?*
- *You were unable to eat healthy and nutritious food?*
- *You ate only a few kinds of foods?*
- *You had to skip a meal?*
- *You ate less than you thought you should?*
- *Your household ran out of food?*
- *You were hungry but did not eat?*
- *You went without eating for a whole day?*

These questions came from the Food Insecurity Experience Scale.^5^ Sample sizes varied due to item non‐response and ranged from 14 813 (going without food for a whole day) to 15 281 (being hungry but not eating). Rather than present and model one standard measure of food insecurity, our modelling presents multiple discrete measures and composite measures of food insecurity as indicators of the sensitivity of individual health conditions to the relationship with food insecurity.

For each measure of food insecurity, prevalence in the population according to specific health conditions was estimated. For each condition, the average number of food insecurity problems (0–8) was also estimated. Binary logistic regression models for each measure of food insecurity estimated associations between each health condition and food insecurity while controlling for various demographic and socioeconomic variables: age, age squared, female, household type, (couple without children, couple with children, lone parent, lone person, other), education, employment, home ownership, income, living in urban areas and social interactions. The controls captured economic and social resources alongside demographic factors in explaining food insecurity. Potential control variables that may be highly correlated with individual health conditions were not included in the analyses due to multicollinearity concerns.

To complement the logistic regression analyses, the association of health conditions with multiple food insecurity events using a count regression process was also examined. Food insecurity is a skewed event, with the majority of respondents reporting food security. Given the high share of zeros in the data, a zero‐inflated negative binomial model was estimated on numbers of problems using a count process. A zero‐inflated negative binomial model contains a binary component that identifies any food insecurity, and a count process for the number of food insecurity problems conditional on any food insecurity being observed. All analysis was performed using Stata 18.

This cross‐sectional study adheres to the STROBE reporting guidelines and ethics approval for this project was granted by The University of Melbourne Human Ethics Committee LNR 2D—Ethics ID 2022‐24371‐31475‐4. All methods were performed in accordance with the relevant guidelines and regulations.

## 3 RESULTS

Our population‐weighted survey data indicate the prevalence of food insecurity in the Australian adult population ranged from 3.06% (running out of food) to 8.86% (ate only a few kinds of foods) (Table 1). This equates to between 620 000 and 1.8 million adults reporting food insecurity in 2020. Considering each of the reported health conditions, excluding hearing problems and, on some food insecurity items, speech problems, individuals with any condition were significantly more likely to report food insecurity than those who did not have the condition. Comparing individuals without each condition to those with each condition, conditions associated with the largest discrepancy in food insecurity were blackouts, fits or loss of consciousness (about 5–12 times the rate of food insecurity), a nervous or emotional condition which requires treatment (about 3–5 times the rate of food insecurity), any disfigurement or deformity (about 3–6 times the rate of food insecurity), any mental illness which requires help or supervision (about 3–5 times the rate of food insecurity) and long‐term effects as a result of a head injury, stroke or other brain damage (about 3–5 times the rate of food insecurity).

**TABLE 1:** Population health conditions and food insecurity, 2020.

Health condition		Food insecurity measure^a^, ^b^								% of category	Number of problems^b^	Number of problems >0
		1	2	3	4	5	6	7	8			
		%	%	%	%	%	%	%	%			
Sight problems not corrected by glasses/lenses	No	5.67	7.15	8.66	4.97	6.24	3.02	5.30	3.70	97.83	0.45	3.35
	Yes	9.69**	11.96**	17.78***	8.97**	13.33***	5.06*	9.41**	10.33***	2.17	0.86***	3.57
Hearing problems	No	5.76	7.24	8.74	5.00	6.30	3.08	5.35	3.79	95.74	0.45	3.36
	Yes	5.57	7.62	11.50	6.43	8.64*	2.73	6.13	4.97	4.26	0.53	3.29
Speech problems	No	5.72	7.18	8.79	5.01	6.34	3.04	5.37	3.82	99.27	0.45	3.35
	Yes	10.78	17.24*	18.90*	12.07	14.08*	5.60	7.87	6.53	0.73	0.93*	3.8
Blackouts, fits or loss of consciousness	No	5.52	7.01	8.62	4.84	6.19	2.92	5.18	3.59	99.32	0.44	3.3
	Yes	40.81***	43.81***	46.24***	38.95***	37.96***	25.11***	36.83***	41.58***	0.68	3.09***	5.51***
Difficulty learning or understanding things	No	5.59	7.11	8.60	4.91	6.18	2.93	5.21	3.70	98.01	0.44	3.33
	Yes	14.14***	14.49***	22.28***	12.68***	16.95***	9.62***	14.01***	10.95***	1.99	1.15***	4.02**
Limited use of arms or fingers	No	5.48	7.00	8.41	4.84	6.05	2.93	5.19	3.67	96.50	0.43	3.33
	Yes	13.36***	14.27***	21.31***	10.98***	15.99***	6.68***	10.80***	8.59***	3.50	1.02***	3.66
Difficulty gripping things	No	5.46	7.02	8.42	4.85	6.08	2.92	5.22	3.71	96.36	0.43	3.35
	Yes	13.50***	13.43***	20.58***	10.61***	14.79***	6.75***	9.83***	7.43***	3.64	0.97***	3.4
Limited use of feet or legs	No	5.46	6.91	8.39	4.72	6.04	2.88	5.09	3.57	94.76	0.43	3.31
	Yes	11.12***	13.46***	17.44***	11.12***	12.79***	6.33***	10.70***	8.76***	5.24	0.91***	3.80*
A nervous or emotional condition which requires treatment	No	4.94	6.54	7.92	4.35	5.67	2.67	4.69	3.26	95.17	0.40	3.22
	Yes	21.69***	21.30***	27.48***	18.97***	20.59***	10.77***	19.06***	15.21***	4.83	1.54***	4.26***
Any condition that restricts physical activity or physical work (e.g., back problems, migraines)	No	4.89	6.34	7.77	4.30	5.50	2.54	4.61	3.18	89.57	0.39	3.2
	Yes	13.11***	15.01***	18.21***	11.55***	14.00***	7.51***	12.00***	9.43***	10.43	1.01***	4.01***
Any disfigurement or deformity	No	5.70	7.16	8.75	4.98	6.32	3.02	5.31	3.74	99.38	0.45	3.34
	Yes	14.67*	22.91***	27.41***	17.62**	19.04**	10.29*	17.70***	20.94***	0.62	1.50***	4.58**
Any mental illness which requires help or supervision	No	5.13	6.70	8.25	4.57	5.91	2.71	4.84	3.47	96.95	0.41	3.25
	Yes	25.56***	24.60***	28.24***	20.65***	21.93***	14.16***	22.83***	15.70***	3.05	1.73***	4.46***
Shortness of breath or difficulty breathing	No	5.47	6.86	8.45	4.76	6.02	2.88	5.10	3.54	96.14	0.43	3.3
	Yes	12.92***	16.92***	19.27***	12.58***	15.78***	7.72***	12.65***	11.38***	3.86	1.09***	4.09***
Chronic or recurring pain	No	5.07	6.48	7.86	4.42	5.58	2.67	4.72	3.25	91.41	0.40	3.24
	Yes	12.99***	15.35***	19.41***	11.79***	14.99***	7.21***	12.45***	9.97***	8.59	1.04***	3.91***
Long‐term effects as a result of a head injury, stroke or other brain damage	No	5.56	7.03	8.60	4.85	6.18	2.93	5.18	3.62	98.54	0.44	3.3
	Yes	18.60***	22.30***	26.37***	18.83***	20.68***	12.08***	19.36***	18.74***	1.46	1.57***	5.22***
A long‐term condition or ailment which is still restrictive even though it is being treated or medication being taken for it	No	5.21	6.65	8.08	4.50	5.78	2.80	4.95	3.41	91.77	0.41	3.28
	Yes	11.82***	13.97***	17.56***	11.26***	13.26***	5.97***	10.23***	8.56***	8.23	0.92***	3.80***
Any other long‐term condition such as arthritis, asthma, heart disease, Alzheimer's disease, dementia, etc.	No	5.22	6.66	8.03	4.50	5.82	2.81	4.96	3.42	87.06	0.41	3.27
	Yes	9.34***	11.25***	14.48***	8.83***	10.26***	4.74***	8.27***	6.66***	12.94	0.74***	3.72**
Population		5.75	7.25	8.86	5.06	6.39	3.06	5.38	3.84	100.00	0.45	3.36
95% CI		5.18, 6.32	6.62, 7.87	8.11, 9.61	4.50, 5.61	5.78, 7.01	2.62, 3.50	4.81, 5.95	3.36, 4.33			

^a^ Food insecurity measures: (1) You were worried you would not have enough food to eat (sample 15 267, missing 22); (2) You were unable to eat healthy and nutritious food (sample 15 260, missing 29); (3) You ate only a few kinds of foods (sample 15 264, missing 25); (4) You had to skip a meal (sample 15 269, missing 20); (5) You ate less than you thought you should (sample 15 267, missing 22); (6) Your household ran out of food (sample 15 267, missing 22); (7) You were hungry but did not eat (sample 15 281, missing 8); (8) You went without eating for a whole day (sample 14 813, missing 476).

^b^ Wald test of difference in food insecurity measure from first category and difference in number of problems compared to first category for each variable: *p, 0.1; **p < 0.05; ***p < 0.01. *n* = 15 463. *N* = 20 289 842.

All conditions except hearing and speech problems showed significant differences in numbers of food insecurity problems when comparing individuals with and without each condition: having each condition was associated with more problems. This result echoed that of the increased prevalence of food insecurity for each item when comparing individuals with and without these conditions. Interestingly, when examining the number of food insecurity problems among individuals with at least one health condition, the number of problems was not significantly different for sight, hearing and speech conditions, limited use of arms or fingers and difficulty gripping things when comparing those with and without the condition. For sight conditions and limitations associated with arms or fingers it seems differences in food insecurity are driven by having food insecurity or not, rather than the ‘depth of disadvantage’ (number of problems). Among those reporting some form of food insecurity, those with the highest number of problems were those reporting blackouts, fits or loss of consciousness (5.51 problems) and those with long‐term effects as a result of a head injury, stroke or other brain damage (5.22 problems), compared to a population average of 3.36 problems.

Table 2 indicates the association between each health condition and food insecurity of each type, controlling for other health and sociodemographic characteristics. Individuals suffering from sight problems not corrected by glasses or lenses were 1.4 times more likely to report eating a few kinds of foods and 1.85 times more likely to report going without food for a whole day compared to other individuals. Individuals with speech problems were 0.23 times less likely to report going hungry and 0.17 times less likely to go without food for a whole day. Individuals suffering from blackouts, fits or loss of consciousness were 2.6–6.2 times more likely to report food insecurity on any item compared to other individuals. Those with difficulty learning or understanding things were 0.6 times less likely to report eating healthy foods than those without such difficulties. Having limited use of feet or legs was associated with almost twice the likelihood of skipping meals, going hungry or going without food for a whole day. Apart from running out of food (which was not significant), it was 1.3–1.7 times more likely for those with a nervous or emotional condition which required treatment to have any other type of food insecurity. Having any condition that restricts physical activity or physical work (e.g., back problems, migraines) was associated with being 1.4–2.0 times more likely than those without such conditions on any measure of food insecurity apart from eating a few kinds of foods, which was not significant. Those with a disfigurement or deformity were 2.2 times more likely to go without food for a whole day. Having a mental illness meant being 1.4–1.9 times more likely of experiencing food insecurity on most measures. Those with shortness of breath of difficulty breathing were more likely of being unable to eat healthy foods (1.5), eating less than they thought they should (1.4) and going without food for a whole day (1.6). Compared to those not reporting pain, having chronic/recurring pain was associated with being more likely to eat few foods (1.4), eating less that they thought they should (1.5), going hungry (1.5) and not eating for a whole day (1.6). Those reporting long‐term effects as a result of a head injury, stroke or other brain damage were more likely to experience food insecurity on every measure. Individuals having other long‐term conditions, such as arthritis, asthma, heart disease, Alzheimer's disease, dementia, were 1.5 times more likely to skip a meal.

**TABLE 2:** Food insecurity logit modelling odds ratios (population weighted), selected results 2020.

Food insecurity measure^a^	1		2		3		4		5		6		7		8	
	OR	95% CI	OR	95% CI	OR	95% CI	OR	95% CI	OR	95% CI	OR	95% CI	OR	95% CI	OR	95% CI
Health conditions																
Sight problems not corrected by glasses/lenses	1.03	(0.60, 1.76)	1.04	(0.62, 1.76)	1.40*	(0.94, 2.07)	0.98	(0.56, 1.73)	1.43	(0.89, 2.29)	0.89	(0.44, 1.80)	1.18	(0.59, 2.35)	1.85*	(0.94, 3.62)
	1.80***	(*1.23*, *2.62*)	1.78***	(*1.19*, *2.66*)	2.34***	(*1.67*, *3.27*)	1.90***	(*1.26*, *2.87*)	2.34***	(*1.61*, *3.41*)	1.65*	(*0.96*, *2.83*)	1.86***	(*1.21*, *2.87*)	3.01***	(*1.95*, *4.63*)
Hearing problems	0.88	(0.52, 1.47)	0.86	(0.52, 1.43)	1.09	(0.73, 1.61)	1.19	(0.72, 1.98)	1.27	(0.83, 1.95)	0.74	(0.35, 1.60)	1.18	(0.69, 2.01)	1.1	(0.61, 1.99)
	*0.97*	(*0.63*, *1.48*)	*1.07*	(*0.70*, *1.63*)	1.38*	(*0.99*, *1.93*)	*1.33*	(*0.88*, *1.99*)	1.42*	(*0.99*, *2.05*)	*0.85*	(*0.46*, *1.57*)	*1.16*	(*0.75*, *1.79*)	*1.31*	(*0.80*, *2.14*)
Speech problems	0.38	(0.11, 1.32)	0.92	(0.35, 2.46)	0.62	(0.27, 1.41)	0.56	(0.17, 1.80)	0.51	(0.19, 1.39)	0.39	(0.10, 1.53)	0.23**	(0.07, 0.77)	0.17**	(0.04, 0.68)
	*1.85*	(*0.77*, *4.40*)	2.78***	(*1.34*, *5.77*)	2.52***	(*1.27*, *5.03*)	2.74**	(*1.20*, *6.24*)	2.52**	(*1.17*, *5.42*)	*1.96*	(*0.73*, *5.32*)	*1.57*	(*0.63*, *3.88*)	*1.82*	(*0.61*, *5.45*)
Blackouts, fits or loss of consciousness	4.13***	(2.00, 8.53)	3.33***	(1.77, 6.29)	3.03***	(1.61, 5.69)	4.08***	(2.04, 8.15)	2.60**	(1.26, 5.40)	3.45***	(1.37, 8.69)	3.30***	(1.62, 6.72)	6.24***	(3.34, 11.66)
	11.95***	(*6.60*, *21.64*)	10.31***	(*5.68*, *18.72*)	9.29***	(*5.30*, *16.27*)	12.81***	(*6.89*, *23.80*)	9.32***	(*4.96*, *17.53*)	10.98***	(*4.83*, *24.94*)	10.74***	(*5.58*, *20.68*)	19.28***	(*10.38*, *35.78*)
Difficulty learning or understanding things	0.64	(0.37, 1.13)	0.61*	(0.35, 1.07)	1	(0.65, 1.55)	0.69	(0.38, 1.23)	0.88	(0.54, 1.43)	0.9	(0.44, 1.85)	0.68	(0.39, 1.19)	0.76	(0.41, 1.38)
	2.76***	(*1.79*, *4.27*)	2.27***	(*1.48*, *3.49*)	3.13***	(*2.18*, *4.48*)	2.92***	(*1.87*, *4.55*)	3.17***	(*2.15*, *4.66*)	3.62***	(*2.05*, *6.37*)	3.04***	(*2.00*, *4.62*)	3.27***	(*2.07*, *5.16*)
Limited use of arms or fingers	1.05	(0.61, 1.82)	0.91	(0.60, 1.38)	1.18	(0.81, 1.73)	0.89	(0.53, 1.52)	1.26	(0.75, 2.11)	0.84	(0.45, 1.58)	0.96	(0.51, 1.79)	0.88	(0.47, 1.67)
	2.72***	(*1.88*, *3.94*)	2.23***	(*1.67*, *3.00*)	3.03***	(*2.27*, *4.05*)	2.49***	(*1.75*, *3.54*)	3.02***	(*2.14*, *4.26*)	2.37***	(*1.56*, *3.58*)	2.25***	(*1.60*, *3.16*)	2.47***	(*1.69*, *3.63*)
Difficulty gripping things	1.47	(0.81, 2.67)	0.98	(0.62, 1.55)	1.22	(0.79, 1.87)	0.99	(0.59, 1.66)	1.14	(0.67, 1.95)	1.25	(0.62, 2.53)	0.86	(0.44, 1.67)	0.65	(0.33, 1.29)
	2.79***	(*1.94*, *4.02*)	2.11***	(*1.56*, *2.86*)	2.91***	(*2.18*, *3.89*)	2.43***	(*1.75*, *3.38*)	2.76***	(*1.98*, *3.85*)	2.48***	(*1.63*, *3.78*)	2.04***	(*1.47*, *2.83*)	2.14***	(*1.48*, *3.09*)
Limited use of feet or legs	1.42	(0.82, 2.47)	1.4	(0.90, 2.16)	1.19	(0.79, 1.80)	1.86**	(1.10, 3.16)	1.29	(0.80, 2.07)	1.6	(0.79, 3.23)	1.84**	(1.10, 3.09)	1.79**	(1.03, 3.12)
	2.24***	(*1.64*, *3.06*)	2.21***	(*1.63*, *2.99*)	2.41***	(*1.84*, *3.15*)	2.67***	(*1.90*, *3.75*)	2.40***	(*1.79*, *3.21*)	2.39***	(*1.55*, *3.67*)	2.37***	(*1.72*, *3.26*)	2.73***	(*1.87*, *3.99*)
A nervous or emotional condition which requires treatment	1.69***	(1.23, 2.31)	1.34**	(1.01, 1.78)	1.60***	(1.23, 2.08)	1.49**	(1.08, 2.06)	1.41**	(1.03, 1.94)	1.19	(0.76, 1.87)	1.49**	(1.09, 2.05)	1.63***	(1.17, 2.29)
	5.55***	(*4.37*, *7.04*)	4.00***	(*3.20*, *5.00*)	4.50***	(*3.59*, *5.63*)	5.07***	(*3.93*, *6.54*)	4.45***	(*3.48*, *5.68*)	4.56***	(*3.34*, *6.23*)	5.00***	(*3.98*, *6.29*)	5.59***	(*4.31*, *7.24*)
Any condition that restricts physical activity or physical work (e.g., back problems, migraines)	1.64**	(1.11, 2.43)	1.51**	(1.09, 2.08)	1.26	(0.93, 1.71)	1.50*	(0.97, 2.32)	1.44*	(0.99, 2.09)	2.00**	(1.12, 3.58)	1.73***	(1.15, 2.60)	1.60*	(0.98, 2.60)
	3.01***	(*2.42*, *3.74*)	2.69***	(*2.20*, *3.28*)	2.69***	(*2.23*, *3.24*)	2.97***	(*2.36*, *3.75*)	2.89***	(*2.35*, *3.55*)	3.21***	(*2.36*, *4.38*)	2.93***	(*2.35*, *3.65*)	3.26***	(*2.54*, *4.19*)
Any disfigurement or deformity	0.64	(0.26, 1.57)	1.28	(0.67, 2.45)	1.14	(0.61, 2.15)	0.98	(0.39, 2.47)	0.88	(0.40, 1.91)	1.13	(0.46, 2.79)	1.04	(0.44, 2.48)	2.20*	(0.87, 5.53)
	2.99***	(*1.45*, *6.19*)	4.01***	(*2.18*, *7.39*)	4.16***	(*2.30*, *7.53*)	4.34***	(*2.17*, *8.67*)	3.66***	(*1.89*, *7.10*)	3.87***	(*1.72*, *8.67*)	4.04***	(*2.12*, *7.72*)	7.18***	(*3.72*, *13.85*)
Any mental illness which requires help or supervision	1.87***	(1.28, 2.73)	1.58**	(1.11, 2.25)	1.31	(0.92, 1.87)	1.41*	(0.95, 2.10)	1.22	(0.81, 1.84)	1.69*	(0.99, 2.87)	1.64**	(1.12, 2.41)	1.07	(0.68, 1.70)
	6.36***	(*4.88*, *8.29*)	4.66***	(*3.53*, *6.16*)	4.40***	(*3.29*, *5.87*)	5.48***	(*4.09*, *7.33*)	4.45***	(*3.33*, *5.95*)	6.02***	(*4.22*, *8.59*)	5.90***	(*4.52*, *7.70*)	5.17***	(*3.70*, *7.23*)
Shortness of breath or difficulty breathing	1.2	(0.77, 1.86)	1.52**	(1.06, 2.18)	1.14	(0.80, 1.62)	1.32	(0.86, 2.01)	1.43*	(0.96, 2.14)	1.42	(0.86, 2.34)	1.46	(0.90, 2.36)	1.55*	(0.97, 2.49)
	2.62***	(*1.91*, *3.60*)	2.83***	(*2.11*, *3.78*)	2.67***	(*2.06*, *3.45*)	2.96***	(*2.16*, *4.05*)	2.99***	(*2.22*, *4.03*)	2.82***	(*1.88*, *4.22*)	2.75***	(*1.96*, *3.85*)	3.52***	(*2.42*, *5.12*)
Chronic or recurring pain	1.14	(0.76, 1.70)	1.33*	(0.95, 1.86)	1.36*	(0.96, 1.93)	1.25	(0.81, 1.91)	1.54**	(1.07, 2.22)	1.22	(0.71, 2.07)	1.48**	(1.00, 2.17)	1.55**	(1.00, 2.41)
	2.86***	(*2.29*, *3.57*)	2.71***	(*2.19*, *3.35*)	2.89***	(*2.36*, *3.54*)	3.01***	(*2.37*, *3.82*)	3.09***	(*2.50*, *3.83*)	2.92***	(*2.15*, *3.95*)	2.98***	(*2.39*, *3.72*)	3.39***	(*2.61*, *4.41*)
Long‐term effects as a result of a head injury, stroke or other brain damage	2.02*	(0.92, 4.47)	1.88*	(0.99, 3.57)	1.83*	(0.99, 3.36)	2.20**	(1.01, 4.75)	1.98*	(0.94, 4.15)	2.07*	(0.98, 4.35)	2.82***	(1.29, 6.15)	3.10***	(1.45, 6.64)
	3.98***	(*2.35*, *6.73*)	3.81***	(*2.31*, *6.29*)	3.87***	(*2.40*, *6.25*)	4.59***	(*2.66*, *7.93*)	3.96***	(*2.33*, *6.76*)	4.41***	(*2.35*, *8.26*)	4.40***	(*2.50*, *7.74*)	6.05***	(*3.47*, *10.54*)
A long‐term condition or ailment which is still restrictive even though it is being treated or medication being taken for it	0.93	(0.67, 1.28)	1.01	(0.76, 1.34)	1.03	(0.77, 1.37)	1.04	(0.75, 1.45)	1.07	(0.79, 1.47)	0.76	(0.49, 1.17)	0.84	(0.58, 1.21)	0.91	(0.62, 1.35)
	2.46***	(*1.97*, *3.06*)	2.30***	(*1.87*, *2.83*)	2.42***	(*1.98*, *2.96*)	2.67***	(*2.10*, *3.38*)	2.51***	(*2.02*, *3.12*)	2.18***	(*1.59*, *2.98*)	2.21***	(*1.72*, *2.85*)	2.69***	(*2.09*, *3.48*)
Any other long‐term condition such as arthritis, asthma, heart disease, Alzheimer's disease, dementia, etc.	1.25	(0.90, 1.75)	1.19	(0.89, 1.59)	1.25	(0.95, 1.64)	1.46**	(1.04, 2.06)	1.13	(0.84, 1.54)	1.15	(0.72, 1.85)	1.22	(0.86, 1.74)	1.3	(0.84, 2.00)
	1.86***	(*1.48*, *2.33*)	1.77***	(*1.46*, *2.15*)	1.97***	(*1.63*, *2.38*)	2.07***	(*1.61*, *2.65*)	1.86***	(*1.50*, *2.31*)	1.74***	(*1.26*, *2.41*)	1.72***	(*1.37*, *2.17*)	2.02***	(*1.51*, *2.69*)
Controls	Yes		Yes		Yes		Yes		Yes		Yes		Yes		Yes	

^a^ Food insecurity measures: (1) You were worried you would not have enough food to eat; (2) You were unable to eat healthy and nutritious food; (3) You ate only a few kinds of foods; (4) You had to skip a meal; (5) You ate less than you thought you should; (6) Your household ran out of food; (7) You were hungry but did not eat; (8) You went without eating for a whole day. Unadjusted results in italics. Control variables were: age, age squared, female, household type (couple without children, couple with children, lone parent, lone person, other), education, employment, home ownership, income, living in urban areas and social interactions. We tested for a range of non‐linear effects in our modelling regime. Our analyses showed a non‐linear relationship between age and food insecurity measures and thus square terms were included in the analyses. We tested alternative model specifications, but the underlying conclusion that health conditions were associated with food insecurity was maintained. *p, 0.1; **p < 0.05; ***p < 0.01.

Table 3 shows results of the ZINB model, in which we modelled the probability of any food insecurity and the number of food insecurity problems. Unlike the figures in Table 1, these results control for the effects of other conditions and control variables. In conducting such a multivariable analysis, we see some differences from the single variable analysis in Table 1. When including control variables and incorporating other health conditions, we see that having sight problems, blackouts, a nervous condition, mental illness or pain was associated with a lower probability of not having any food insecurity (i.e., increased probability of any food insecurity) but with no significant effect on the expected number of food insecurity problems. Difficulty gripping things was associated with increased probability of food insecurity but a reduced expected number of food insecurity problems. Although the probability of food insecurity was not significant, the expected number of problems was higher if individuals had limited use of legs, a condition that restricts physical activity, long‐term effects of head injury, stroke or other brain damage, or other long‐term conditions such as arthritis, asthma, heart disease, Alzheimer's disease or dementia.

**TABLE 3:** Food insecurity zero‐inflated negative binomial modelling (population weighted), selected results 2020.

Health conditions	Negative binomial model	Logit model^a^		Negative binomial model	Logit model^a^
	IRR	Coef.		IRR	Coef.
Sight problems not corrected by glasses/lenses	0.64** [0.44, 0.95]	−0.10 [−0.32, 0.12]	Any condition that restricts physical activity or physical work (e.g., back problems, migraines)	0.82 [0.64, 1.06]	0.15** [0.02, 0.28]
	(0.13)	(0.11)		(0.11)	(0.07)
Hearing problems	0.97 [0.69, 1.36]	0.04 [−0.18, 0.25]	Any disfigurement or deformity	0.98 [0.54, 1.77]	0.00 [−0.28, 0.28]
	(0.17)	(0.11)		(0.30)	(0.14)
Speech problems	1.75 [0.79, 3.88]	−0.13 [−0.50, 0.24]	Any mental illness which requires help or supervision	0.61*** [0.44, 0.85]	0.00 [−0.14, 0.14]
	(0.71)	(0.19)		(0.10)	(0.07)
Blackouts, fits or loss of consciousness	0.29*** [0.14, 0.59]	0.18 [−0.05, 0.41]	Shortness of breath or difficulty breathing	0.83 [0.59, 1.16]	0.11 [−0.53, 0.15]
	(0.10)	(0.12)		(0.14)	(0.08)
Difficulty learning or understanding things	1.16 [0.75, 1.79]	−0.05 [−0.29, 0.58]	Chronic or recurring pain	0.72** [0.54, 0.98]	0.05 [−0.10, 0.20]
	(0.26)	(0.11)		(0.11)	(0.08)
Limited use of arms or fingers	1.05 [0.72, 1.53]	0.09 [−0.11, 0.29]	Long‐term effects as a result of a head injury, stroke or other brain damage	0.81 [0.45, 1.46]	0.34*** [0.10, 0.57]
	(0.20)	(0.10)		(0.24)	(0.12)
Difficulty gripping things	0.59*** [0.41, 0.87]	−0.23** [−0.45, −0.00]	A long‐term condition or ailment which is still restrictive even though it is being treated or medication being taken for it	0.93 [0.72, 1.20]	0.02 [−0.11, 0.16]
	(0.12)	(0.11)		(0.12)	(0.07)
Limited use of feet or legs	0.87 [0.63, 1.20]	0.20** [0.01, 0.40]	Any other long‐term condition such as arthritis, asthma, heart disease, Alzheimer's disease, dementia, etc.	0.94 [0.74, 1.19]	0.13** [0.00, 0.26]
	(0.14)	(0.10)		(0.11)	(0.06)
A nervous or emotional condition which requires treatment	0.65*** [0.50, 0.85]	0.06 [−0.07, 0.18]			
	(0.09)	(0.06)	Controls	Yes	Yes

^a^ Logit model reflects probability of no food insecurity. SE in parentheses, 95% Confidence Interval in brackets. ln(alpha) −1.14*** (0.14). *n* = 47 622. Control variables were: age, age squared, female, household type (couple without children, couple with children, lone parent, lone person, other), education, employment, home ownership, income, living in urban areas and social interactions. We tested for a range of non‐linear effects in our modelling regime. Our analyses showed a non‐linear relationship between age and food insecurity measures and thus square terms were included in the analyses. We tested alternative model specifications, but the underlying conclusion that health conditions were associated with food insecurity was maintained. *p, 0.1; **p < 0.05; ***p < 0.01.

## 4 DISCUSSION

Despite low prevalence of many of the health conditions studied, most health conditions showed some association with food insecurity of any type. Therefore, although any of these conditions affected less than 5% of the population, there are clearly individuals at high risk of food insecurity. Controlling for other factors associated with food insecurity, findings were that most health conditions were associated with at least one form of food insecurity, with blackouts and fits, and head injuries/stroke significantly associated with all forms of food insecurity. However, hearing problems, limited use of arms/fingers, difficulty gripping things and having a long‐term condition which is restrictive showed no significant association with any form of food insecurity.

Unlike the well described bidirectional relationship between mental health and food security,^24^ to date, there has been limited studies on food insecurity and fits (e.g., Febrile convulsion),^29^ and no studies on food insecurity in relation to experiencing blackouts, or loss of consciousness. Blackouts are particularly interesting in that the direction of causality could go either way: they could either cause food insecurity (if the condition makes individuals less able to get out and source food or have medical expenses that ‘crowd out’ spending on food) or result from food insecurity (if individuals are suffering due to missing meals or running out of food). While the conditions found associated with food insecurity could represent flags for intervention, the current study's data do not permit causal analysis nor the direction of causal analysis. In any case, the relationship may be so complex that it cannot, in practice, be untangled. Many of the other conditions are likely to be long‐term and pre‐date the reported experience of food insecurity, consistent with a causal explanation.

The probability of having any type of food insecurity was higher for those who experienced sight problems, blackouts, fits or loss of consciousness, who had difficulty gripping things, who had a nervous condition, who had mental illness or suffered from chronic pain. Vision impairment has been studied in the context of food insecurity (e.g., Kumar et al.^30^; older adults in six low‐ and middle‐income countries^31^; and among older adults in the USA^25^, ^32^). Similarly, chronic pain has been studied in the context of food insecurity (e.g., including use of prescription opioids^20^; high‐impact chronic pain in the United States^33^; and depressive symptoms among urban food bank users^21^). However, those experiencing blackouts or loss of consciousness, have difficulty gripping things, and who had a nervous condition have not been specifically studied in the context of food insecurity.

The depth of disadvantage, represented by numbers of items on which individuals were food insecure, was higher for individuals with difficulty gripping things, limited use of feet or legs, physical restrictions, with long‐term effects as a result of a head injury, stroke or other brain damage or with other long‐term conditions such as arthritis, asthma, heart disease, Alzheimer's disease or dementia.

The bivariate analysis suggested those with limited use of legs were likely to have an average of 3.8 types of food insecurity problems compared to 3.3 problems for those without this condition. These problems were most likely to be skipping meals, going hungry and going without food for a whole day. Controlling for covariates, this heightened experience of food insecurity still stands. Having physical restrictions also showed consistent results between the analyses: increased numbers of problems that were most likely to be running out of food, going hungry, worrying about having enough food and not eating for a whole day. Head injuries, stroke or other forms of brain damage had higher expected numbers of food insecurity problems in all analyses, although with each measure of food insecurity significantly associated with this condition, there was no clear pattern in types of food insecurity. Previous studies of food insecurity in relation to stroke have included prevalence and predictors in the United States,^34^ financial stress and cost‐related medication non‐adherence.^35^ Other long‐term conditions (arthritis, etc.) were associated with more food insecurity problems than those without these conditions and the extra problem was likely to be skipping meals. Previous studies of food insecurity in relation to arthritis have included their association with depression,^36^ and in relation to other socioeconomic factors.^37^ Unfortunately, the literature does not seem to extend to analysing the depth of disadvantage in food insecurity.

The results on the association of food insecurity and sight problems, nervous/emotional conditions/mental health, asthma/shortness of breath, chronic or recurring pain and stroke or heart disease are consistent with the literature discussed earlier. However, while there is some evidence in the literature linking food insecurity and hearing problems (e.g., Saunders et al.,^38^ who conducted a scoping review with the included quantitative studies using a single item tool, and Gopinath et al.,^26^ who used a 12‐item food security survey), the current research could not find significant associations of this condition with food insecurity. This may be due to studies including a limited set of control variables or none at all. The current study differs and builds on previous studies in the following ways, (i) the results control simultaneously for all nominated conditions in the study and (ii) include a representative population sample that is not limited to specific groups. As such, we do not find some effects as they are specific to certain segments of the population. (iii) We also use a multiple‐item food insecurity tool, which is more comprehensive than a single‐item question.

In interpreting results of the current study, it is important to take note of the timing with Australia reported its first cases of COVID‐19 on 25 January 2020.^25^ The HILDA data used was conducted in 2020 during a time when COVID‐19 pandemic food insecurity likely increased.^26^ In addition, COVID‐19 likely exacerbated long‐term health conditions in recovery from infection, and economic vulnerabilities brought about by unavailability of work (e.g., including sudden and significant loss of employment, underemployment and income reductions).^27^ The impact of government enforced public health measures such as travel‐bans, lockdowns, and social distancing restrictions also negatively impacted access to food.^27^ Food availability was affected by logistic issues of supply^28^ changes in consumer spending and stockpiling,^27^ and Australian supermarkets placing strict limitations on the amount of staple food items that could be purchased in one transaction.^27^ In addition, COVID‐19 exacerbated some pre‐existing interrelated social inequalities, thereby worsening and having a long‐term impact for people living with chronic health conditions, via disruptions to essential healthcare services, and healthcare screening and healthcare seeking and utilisation.^29^, ^30^, ^31^, ^32^, ^33^, ^34^, ^35^ This context is important as the impacts of COVID would have impacted the reporting of both food insecurity and health conditions in the HILDA survey, relative to non‐pandemic periods.

This study had several strengths. First, the data used were population‐representative and not limited to specific groups. Second, the sample was large at approximately 15 000 adult respondents. Third, our data included 17 sets of health conditions—some very specific and some more general—allowing us to obtain a detailed picture of how health conditions were associated with different types of food insecurity. Fourth, eight measures of food insecurity were included in the current study, whereas the literature often uses only one or two items. This allowed us to get a more nuanced picture of food insecurity for those suffering different health conditions.

There were some limitations of the study. In terms of data, some groups were known to be underrepresented, such as Indigenous Australians, people in very remote areas of Australia and people in non‐private dwellings such as nursing homes/residential aged care, jails schools and hospitals. This is a problem with the sample design of HILDA. The data did not permit us to assess causality in the relationship between health conditions and food insecurity, and the relationship is likely to be complex and bi‐directional. Individuals experiencing food insecurity may be subject to multiple disadvantages that were not captured, such as energy poverty^37^ or more broad income poverty. As the data did not identify cancer as a health condition, we were unable to assess this link with food insecurity and this remains an area for future research.

In conclusion, the prevalence of food insecurity ranged from 3% to 9% depending on the measure. Individuals suffering from blackouts, fits or loss of consciousness were 2–6 times more likely to report food insecurity than other individuals. When including control variables and incorporating other health conditions, several conditions significantly increased probability of any food insecurity: sight problems, blackouts, fits or loss of consciousness, difficulty gripping things, nervous conditions, mental illness and chronic or recurring pain.

Understanding the links between food insecurity and health conditions has significant implications for public health. First, knowledge of the links allows public health practitioners to develop targeted interventions to address specific health issues that may be exacerbated by or linked to food insecurity, leading to more effective and relevant programmes. Identifying the health conditions associated with food insecurity helps target vulnerable populations as public health efforts can contribute to reducing health disparities and ensure equitable health outcomes by tailoring interventions to the needs of underserved groups. Understanding the relationship between food insecurity and health conditions can lead to improved nutritional education through public health messages emphasising the role of good nutrition in preventing or managing health conditions. By addressing food insecurity and its impact on health conditions, public health efforts can contribute to reducing healthcare costs through fewer hospitalizations and medical interventions.

The links between food insecurity and health conditions may also inform research priorities and advocacy efforts as public health professionals can use this knowledge to advocate for policy changes that address the root causes of food insecurity. Finally, recognising the multifaceted problem of food insecurity, public health initiatives can combine nutrition education, economic support and community engagement to address the complex challenges posed by food insecurity. Understanding the connections between food insecurity and health conditions therefore allows public health professionals to create more effective, targeted, and holistic interventions.

## AUTHOR CONTRIBUTIONS

All authors made substantial contributions to the conception and design of the work; the acquisition, analysis and interpretation of data and reviewed the paper critically for important intellectual content. All authors approved the version to be published and agree to be accountable for all aspects of the work in ensuring that questions related to the accuracy or integrity of any part of the work are appropriately investigated and resolved. This article uses unit record data from the Household, Income and Labour Dynamics in Australia (HILDA) Survey. The HILDA Project was initiated and funded by the Australian Government Department of Social Services (DSS) and managed by the Melbourne Institute of Applied Economic and Social Research (Melbourne Institute). The findings and views reported in this article, however, are those of the authors and should not be attributed to either DSS or the Melbourne Institute.

## FUNDING INFORMATION

This work was supported by the Australian Research Council's Centre of Excellence in Population Ageing Research (Jane M. Fry and Jeromey B. Temple, grant number CE1101029).

## CONFLICT OF INTEREST STATEMENT

The authors declare no conflicts of interest.

## ETHICS STATEMENT

Ethics approval for this project was granted by The University of Melbourne Human Ethics Committee LNR 2D – Ethics ID 2022‐24371‐31475‐4. All methods were performed in accordance with the relevant guidelines and regulations.

## Data Availability

The data that support the findings of this study are available from the Australian Data Archive https://dataverse.ada.edu.au/dataverse.xhtml?alias=hilda but restrictions apply. The data were used under licence for the current study, and so are not publicly available.
